# Ketamine as an adjuvant in sympathetic blocks for management of central sensitization following peripheral nerve injury

**DOI:** 10.1186/1749-7221-3-22

**Published:** 2008-10-25

**Authors:** Rani A Sunder, Gokul Toshniwal, GP Dureja

**Affiliations:** 1 Asst Professor, Dept of Anaesthesiology, All India Institute of Medical Sciences, New Delhi, India; 2 Former Resident, Dept of Anaesthesiology, All India Institute of Medical Sciences, New Delhi, India; 3 Ex-Professor Dept of Anaesthesiology, All India Institute of Medical Sciences, Currently, Director – Delhi Pain Management Centre, New Delhi, India

## Abstract

Proliferation of NMDA receptors and role of glutamate in producing central sensitization and 'wind up' phenomena in CRPS [complex regional pain syndrome] forms a strong basis for the use of Ketamine to block the cellular mechanisms that initiate and maintain these changes. In this case series, we describe 3 patients of CRPS Type II with debilitating central sensitization, heat/mechano allodynia and cognitive symptoms that we termed 'vicarious pain'. Each of these patients had dramatic relief with addition of Ketamine as an adjuvant to the sympathetic blocks after conventional therapy failed.

**Case Reports:**

All 3 patients suffered gunshot wounds and developed characteristic features of CRPS Type II. Within 2–3 weeks they developed extraterritorial symptoms typical of central sensitization. The generalized mechanical allodynia and debilitating heat allodynia described to be rare in human subjects had life altering affect on their daily life. Case 2 and 3 also described an unusual cognitive phenomenon i.e. visual stimuli of friction would evoke severe pain in the affected limb that we have termed as 'vicarious pain'. They responded positively to sympathetic blocks but the sympatholysis did not bring relief to the heat and mechanical allodynia. Addition of Ketamine 0.5 mg/kg to the sympathetic blocks elicited resulted in marked relief in the allodynia.

**Conclusion:**

Ketamine has a special role in patients with debilitating heat allodynia and positive cognitive symptoms via its action on central pain pathway. As an adjuvant in sympatholytic blocks it has a targeted action without significant neuropsychiatric side effects.

## Introduction

Complex Regional Pain Syndrome [CRPS] is a painful debilitating condition and the diagnosis is based on consensus criteria developed in 1993[1]. Management can be challenging as this disorder is difficult to treat. Treatments modalities include steroids, sympathetic block, oxygen radical scavengers, antidepressants, antiepileptics, opioids, sympathetic blocks, spinal cord stimulation etc. Despite a multitude of treatment modalities, a subgroup of CRPS patients remain refractory to all standard therapies. In these patients, the disease may spread extraterritorially, which results in severe disability. The life altering allodynia and hyperalgesia experienced by these patients and the non availability of proven therapeutic modalities forces the physician to think of unconventional solutions. Currently there is a resurgence of interest in the role of Ketamine in CRPS [2]. Proliferation of NMDA receptors and role of glutamate in producing central sensitization and 'wind up' phenomena in CRPS forms a strong basis for the use of Ketamine to block the cellular mechanisms that initiate and maintain these changes. Both subanesthetic infusions and Ketamine induced coma have been described in the successful management of chronic pain states [3,4]. In this series of three cases of CRPS type II, we describe a new route for administration of Ketamine – as an adjuvant in sympatholytic blocks [stellate ganglion block] to treat neuropathic pain. We also highlight an unusual positive sensory symptom of 'vicarious' pain.

## Case 1

**Figure 1:**
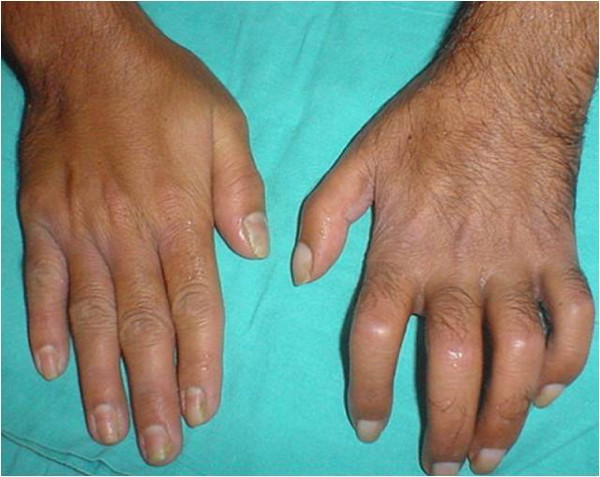
**Typical features of CRPS.** Note the hypertrichosis, edema, joint stiffness nail changes, skin atrophy, mirror image changes in opposite hand.

**Figure 2:**
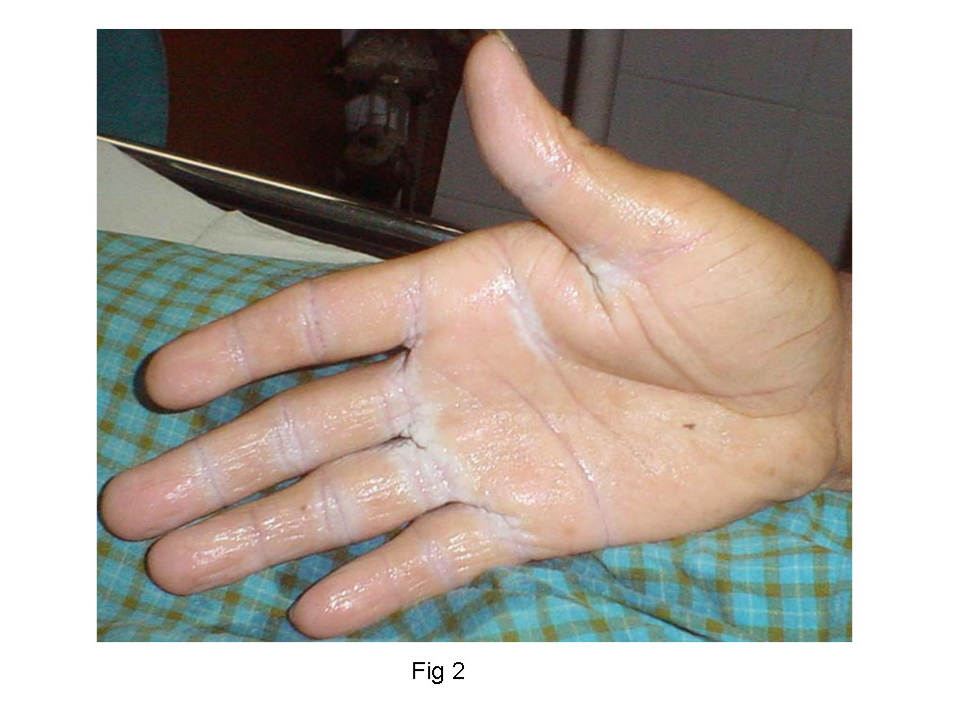
Trophic changes in the opposite hand due to constant exposure to water.

A 30 year old laborer presented with 2 month old history of a gunshot wound [GSW] in his left arm. On initial examination, there was sensory loss in the distribution of medial cutaneus nerve of the forearm without motor deficit. He was managed conservatively. Within 2 days of the injury he developed typical neuropathic pain over his forearm and palm [burning and shock like], thermal and mechanical allodynia, hyperalgesia with edema. Allodynia and edema increased over the next two weeks. There was gradual limitation of interphalyngeal joint movements along with onset of mechanical allodynia in the opposite limb [mirror image symptoms] which later progressed to affect the entire body. Simultaneously, he reported generalized warm allodynia that became progressively so debilitating that he could not step out in the sun. Distressing mechano allodynia prevented him from wearing tight fitting clothes/footwear. He discovered that moistening extremities with cold water reduced allodynia and he resorted to covering his palms and soles with damp towels. When we saw him (4^th ^week after injury), he showed typical characteristics of CRPS Type II [Fig 1] i.e. edema, cold skin, ridging of nails, hypertrichosis, joint stiffness, positive sensory abnormalities and trophic changes in both his extremities [Fig 1, 2] due to constant exposure to water. There was progressive functional impairement of the injured upper limb leading to loss of livelihood. His peak VAS score for evoked pain was 10 and 8 for static pain. Detailed psychiatric evaluation excluded disorders other than depression. He was started on Gabapentin, Tramadol and Amitryptylline and gradually increased to maximum doses. A diagnostic stellate ganglion block produced marked relief. Over the next two weeks he received stellate ganglion blocks [10 ml 0.25% bupivacaine] on alternate days as per institutional protocol. At 8 weeks, VAS for spontaneous pain reduced from 10 to 3 but VAS for evoked pain reduced only marginally (to 7/10). Though he could start regular physiotherapy and was sleeping better, heat allodynia and hyperpathia were still a source of significant distress in the duration of his waking hours [8/10]. At this juncture (9^th ^week), intravenous Lidocaine infusion and SGB with Clonidine as an adjuvant were tried unsuccessfully. In the 10^th ^week, a trial of SGB with 10 mg Ketamine as an adjuvant was given after informed consent and midazolam premedication. He experienced 'light headedness', described as a pleasant floating sensation and reported dynamic VAS score to be 1. The pain relief lasted for 36 hrs. Thereafter, he received 3 days of continuous stellate ganglion infusion [0.0625% Bupivacaine with 0.5 mg Ketamine/day] @ 2 ml/hr. The improvement was rapid, VAS [static] remained 0, dynamic VAS [1,2], with reduction in the intensity of heat and mechanical allodynia [1–2/10]. Weight bearing physiotherapy could be started. The feeling light headedness persisted throughout the period of infusion. He did not report any hallucinations. After discontinuation of infusion [12^th ^week], the patient remained comfortable, continued medications and physiotherapy. He could step out in the sun without a wet towel, wear shoes and lift small weights with his left hand. There was significant improvement in the range of finger movements. At 5 month, 6 month and 1 year reviews he was found to be largely symptom free.

## Case 2

A 45 year old career soldier with history of GSW to his right brachial plexus was referred to us 8 weeks after the injury with history of severe neuropathic symptoms unresponsive to medical management. There was complete motor loss in the right upper limb with sensory loss in the distribution of radial and ulnar nerves. He experienced burning shock like pains in the distribution of the radial nerve with non pitting edema of the affected limb. He experienced severe generalized heat allodynia at temperature above 25°C needing to moisten his palms and face constantly. Distressing mechano allodynia did not permit him to shake hands, sit under the draft of a ceiling fan, wear rough/tight clothing etc. He also had unusual cognitive symptoms pertaining to special senses. The visual stimulus of friction i.e. people rubbing their hands, onscreen action sequences etc would elicit moderate pain in the affected limb. We termed this as 'vicarious pain'. VAS scores at the time of initial interview varied from 7–10 for evoked pain. He was under psychiatric treatment for moderate depression [Beck depression inventory score]. He was already on maximum standard oral pharmacotherapy for neuropathic pain. All treatment modalities were started after discussion with the psychiatrist. SGB with 0.25% 10 ml Bupivacaine [test for sympathetically mediated pain] elicited a good response i.e. reduction in VAS score to 2–3. He was then administered a series of SGB as per institutional protocol described earlier. Despite relief lasting for 4–8 hours, there was no let up in the allodynia and 'vicarious pain' after the block wore out [mean VAS score remained [6]]. He then consented for Ketamine – Bupivacaine SGB infusion after he was explained the experimental nature of the treatment and informed about possible psychomimetic side effects. He received an infusion for 3 days during which he reported mild sedation and euphoria. There were no hallucinations. He experienced reduction in edema scores, mechanical/thermal allodynia and hyperpathia [2/10]. The 'vicarious pain' symptoms also diminished. He continued medical therapy and physiotherapy. He received another 3 day course of Ketamine-Bupivacaine SGB infusion at 6 month after recurrence of allodynia in the injured limb. He was free of troublesome neuropathic symptoms at the end of 1 year He continues to be on oral medications.

## Case 3

A 28 year old male had a 3 month old history of a GSW leading to a foot drop. Within 2 weeks of injury he developed burning pain, allodynia in the distribution of the sciatic nerve and edema of the effected limb. Pharmacotherapy with maximum doses of Gabapentin, Amitryptalline and Tramadol gave him moderate relief [VAS score for evoked pain was [5,6]]. One month after injury he began experiencing extraterritorial symptoms typical of central sensitization i.e. generalized mechanical and thermal allodynia. He also gave history of 'vicarious pain'. Typically, the heat allodynia was relieved by moistening his peripheries. Over the past month thermal and mechanoallodynia were the most distressing daytime symptoms that took a debilitating toll on his personal and professional life. Psychiatric evaluation revealed moderate depression. He reported decrease in evoked pain, to VAS score 3, with a sympatholytic dose of epidural Bupivacaine and was started on epidural infusion of Bupivacaine [0.625% 2 ml/hr after a loading bolus of 10 ml 0.1% Bupivacaine]. The VAS for heat allodynia remained unchanged [5,6], intensity of pain due to mechano-allodynia remained constant at 5. The infusion was stopped on day 5. Morphine and Clonidine were tried as adjuvants to the epidural infusion without success. On day 14, after informed consent a 3 day epidural infusion with preservative free Ketamine, 0.5 mg/kg/24 hrs, was started. He experienced light headedness and mild sedation during the course of therapy. One week after the infusion pain due to heat allodynia and mechanoallodynia were significantly reduced to 0. At 6 month and 1 year review, he described long lasting relief in thermal and 'vicarious' allodynia with ability to resume his duties limited only by the foot drop.

## Discussion

Complex regional pain syndrome [CRPS] is a neuropathic pain syndrome following injury characterized by distal predominance of abnormal findings, and a variable temporal and clinical course. Based on the diagnostic algorithm proposed by IASP all our patients could be categorized under CRPS Type II [5]. All three demonstrated extraterritorial spread of pain and sensory dysfunction characteristic of central sensitization. The unique cognitive symptoms the patients' experienced i.e. perception of burning pain in the affected limb upon visual stimulus of friction is unique to this case series. We termed these symptoms 'vicarious' pain, literally meaning 'endured for another'. From the point of view of lifestyle, dynamic mechanical allodynia is highly disabling for affected subgroups of CRPS patients. Heat allodynia is described to be rare in human subjects was common feature in all our patients [6]. The warm climate of the subcontinent aggravated their suffering.

Most common initiating factor for development of CRPS is trauma leading to initiation of peripheral sensitization and decreased threshold for stimulation of the pain fibers. The increased ectopic activity in the damaged nerve maintains afferent impulse to dorsal root ganglion (DRG) and dorsal horn of spinal cord. These persistent inputs to the dorsal horn of the spinal cord from the C fibers cause "Wind Up" phenomena that produce changes at the synaptic level [7]. A low threshold stimulus like touch is perceived as pain [allodynia]. The change in the type of receptors may either decrease the inhibitory receptors like GABA or increase in the excitatory receptors like NMDA [8]. These plastic changes that increase the sensitivity of the central nervous system occur mainly in the dorsal horn of the spinal cord and may spread to extrasegmental location like contralateral side, above or below the segment as demonstrated by our patients. There is ample evidence of bidirectional immune brain communication and some patients report extraterritorial mirror image pain [9]. Mirror-image allodynia, arises from the healthy body region contralateral to the actual site of trauma/inflammation. Inflammatory cytokines and glia are implicated in this phenomenon. [10]. Data suggests that activation of astrocyte communication via gap junctions may mediate such spread of pain. While traditional therapies for pathological pain have focused on neuronal targets, glia are the newly recognized mediators of exaggerated pain, and new therapeutic targets. Moreover, the glial-neuronal interactions are likely not exclusive to pain, but rather are likely to play significant roles in other behavioral phenomena.

The supraspinal structures involved in maintaining neuropathic pain are rostral ventromedial medulla, para-aqueductal gyrus, amygdale and some cortical areas. Cortical reorganization leads to unmasking of latent synapses, overlap of sensory areas. The extraterritorial spread of symptoms represents cortical reorganization with greater shift in representation of the affected limb [11]. This can explain the abnormal sensory response of 'vicarious pain'. The 'vicarious pain' in our patients cannot be classified as referred pain as they experienced the sensory symptoms when their eyes were open. This phenomenon cannot be classified as dysynchiria as the stimulus evoking pain was through the special senses [visual] and not touch.

NMDA receptors are ubiquitously present in the pain pathway including peripheral tissues and show increased activity in patients presenting with neuropathic symptoms. Specific activation of a medial thalamic pathway to the frontal lobe has been demonstrated with heat allodynia [12]. The thalamocortical dissociation affected by Ketamine may have a role to play in its therapeutic action. All the patients in this case series had heat allodynia and responded to sympatholysis with Ketamine. The effect of opioids appears to be decreased in patients with neuropathic pain [13]. Ketamine on the other hand has been effective after onset of hyperalgesic symptoms [8]. There are changes in the ion channels especially Na^+ ^and Ca^2+ ^channels in neuropathic pain. Therefore combination of a Na^+^channel blocker [local anesthetic] and NMDA receptor antagonists appear to be a rational approach for treatment of neuropathic pain [14].

The role of sympathetic nervous system in the pathophysiology of neuropathic pain is not clear. Abnormal contact develops between the sympathetic nervous system and the sensory system following peripheral nerve injury. Most likely site of coupling is at the dorsal root ganglion [15]. Therefore, sympathetic blockade has a role in CRPS. Patients who respond to sympathetic blocks are categorized to have sympathetically mediated pain (SMP). Sympathetic block is shown to be effective in patients with mechanical and/or cold allodynia and high sensitivity scores on the Neuropathic pain scale score [16]. Our patients exhibited SMP along with heat and mechanical allodynia.

**Figure 3:**
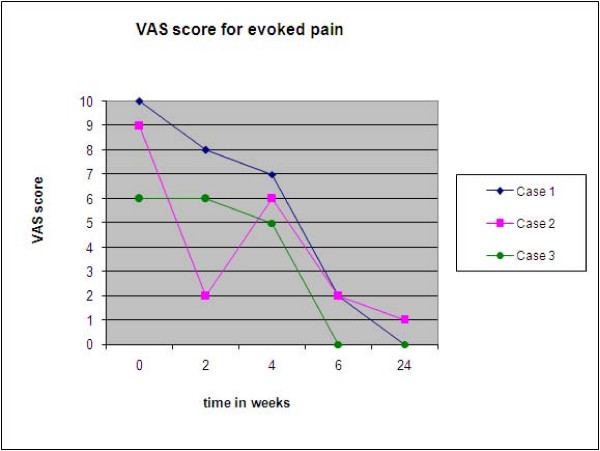
Change in evoked VAS score with time.

Ketamine has been administered orally, as an ointment, intravenously, subcutaneously, epidurally and intramuscularly in a number of case reports in literature[17-19]. Ketamine doses for neuropathic pain reported in literature have ranged from 0.1 mg/kg-7 mg/kg and infusion time ranging from 30 minutes to 8 hours in both CRPS Type I/II patients [7,17,20]. Though there are no randomized controlled trials available in a 2007 open label trial Keifer et al used anesthetic doses of Ketamine in 20 refractory CRPS patients to obtain pain reduction and better quality of life [20]. Later this year he used S (+) Ketamine in refractory CRPS only to abandon the trial due to lack of therapeutic response [21]. Though dose dependent side effects are expected with Ketamine infusions there have been no adverse cognitive effects of extended treatment in CRPS patients [22] We chose a dose of 0.5 mg/kg/24 hours as the sites of injection [stellate ganglion/epidural] were presumed areas of neuronal reorganization and therefore target areas. The initial test dose with 0.5 mg/kg produced good results with minimal psychomimetic effects, so we chose that dose for all our cases. There was rapid amelioration of symptoms in all our patients timed with introduction of Ketamine to the sympathetic blocks [Fig 3].

Techniques to test the effects of therapy on mechanical/thermal allodynia can be expensive and cumbersome in clinical practice. QST [Qualitative Sensory Testing] besides being time consuming depends on expensive equipment and is not specific for neuropathic pains. We used simple brushes, thermorollers and weighted needles recommended in literature to study the various components of the neuropathic pain [23].

The management of neuropathic pain is multimodal and should be mechanism targeted. The action of Ketamine in the sympatholytic block is perhaps multimodal, supraspinal action by systemic absorption and peripheral action through NMDA receptors located either on the somatic nerve or in the dorsal root ganglion. Blockade of peripherally located NMDA receptors is a potential target in the management of neuropathic pain and should be introduced early in therapy. It appears to be an effective route of administering Ketamine with few potential psychomimetic effects. The degree of reduction in allodynia and prolonged relief after introduction of ketamine in all our patients emphasizes the role of NMDA antagonists in reorganization of plastic changes at the peripheral, spinal cord and cortical level. Therefore, we can conclude that Ketamine has a role in patients with debilitating heat allodynia and positive cognitive symptoms.

## Consent Statement

Written informed consent was obtained from the patient for publication of this case report and accompanying images. A copy of the written consent is available for review by the Editor-in-Chief of this journal.

## Competing interests

The authors wish to declare that they have no competing interests.
